# Altered Microglia-Neuron Crosstalk and Regional Heterogeneity in Alzheimer’s Disease Revealed by Single-Nucleus RNA Sequencing

**DOI:** 10.3390/ijms27031492

**Published:** 2026-02-03

**Authors:** Zhenqi Yang, Mingzhao Zhang, Weijia Zhi, Lizhen Ma, Xiangjun Hu, Yong Zou, Lifeng Wang

**Affiliations:** Beijing Institute of Radiation Medicine, 27 Taiping Road, Beijing 100850, China

**Keywords:** Alzheimer’s disease, microglial subtype, microglia-neuron crosstalk, snRNA-seq, THY1

## Abstract

Alzheimer’s disease (AD) is a progressive neurodegenerative disorder characterized by irreversible cognitive decline and synaptic dysfunction and represents the most prevalent etiology of dementia, accounting for an estimated 60–70% of all clinically diagnosed cases worldwide. The growing focus on microglia–neuron interactions in AD research highlights their diverse, region-specific responses, which are driven by the functional and pathological heterogeneity across different brain regions. Therefore, investigating the interactions between microglia and neurons is of crucial importance. To explore the regional heterogeneity of microglia–neuron crosstalk in AD, we integrated human single-nucleus RNA sequencing data from the prefrontal cortex (PFC), hippocampus (HPC), and occipital lobe (OL) provided by the ssREAD database. Our study delineated four microglial subtypes and uncovered a pseudotime trajectory activation trajectory leading to the disease-associated microglia (DAM) phenotype. The transition along this trajectory is driven and stabilized by a key molecular switch: the coordinated downregulation of inhibitory factors (e.g., LINGO1) and upregulation of immune-effector and antigen-presentation programs, which collectively establish the pro-inflammatory DAM state. Furthermore, we observed that each brain region displayed unique microglia–neuron communication patterns in response to AD pathology. The PFC and OL engage a THY1-ITGAX/ITGB2 signaling axis; the HPC predominantly utilizes the PTPRM pathway. Notably, THY1 dysregulation strongly correlates with pathology in the PFC, HPC, and OL, suggesting that microglia–neuron crosstalk in AD possesses both heterogeneity and commonality. The main contribution of this study is the systematic characterization of region-specific microglia-neuron interactions and the identification of THY1 as a potential mediator that may be targeted therapeutically to modulate microglial function in affected brain regions.

## 1. Introduction

Alzheimer’s disease (AD) is the most prevalent neurodegenerative disorder worldwide, affecting over 50 million individuals, and poses a substantial and growing burden on global healthcare systems [1]. The defining neuropathological features of AD include the deposition of amyloid-β (Aβ) plaques and the accumulation of neurofibrillary tangles composed of hyperphosphorylated Tau protein [2,3,4]. The aberrant aggregation of these pathological proteins triggers widespread neuroinflammation in the brain, primarily mediated by hyperactivated microglia [5,6]. The inflammatory response significantly contributes to synaptic dysfunction and neuronal loss, ultimately leading to irreversible brain atrophy and progressive cognitive decline [7,8,9]. The pathogenesis of AD is highly complex, involving multiple cellular mechanisms and pathways that cannot be explained by any single molecular hypothesis [10,11,12,13,14]. Consequently, therapeutic strategies developed over the past decades, largely rooted in the amyloid cascade hypothesis [15], have frequently encountered setbacks in clinical trials [16,17], highlighting the need for a more nuanced understanding of cell-type-specific responses and intercellular interactions [18,19,20]. Microglia transform moderate inflammatory activation to an excessive inflammatory response in AD [21,22,23,24,25,26], while also exhibiting intricate interactions with astrocyte and neurons [27,28,29,30,31,32,33]. These findings indicate that the pathogenesis of AD can alter microglia–neuron crosstalk.

AD neuropathological progression does not occur uniformly across the brain but exhibits remarkable regional specificity and cellular heterogeneity [34,35,36]. Classical studies have established that neurofibrillary tangles (NFTs) typically first appear in the entorhinal cortex before sequentially spreading to the hippocampus, limbic system, and neocortex [37,38]. Similarly, Aβ plaques show distinct accumulation gradients across brain regions, indicating significant differences in regional susceptibility [39,40,41]. Even within the same region, different neuronal and glial cell types exhibit divergent molecular responses and vulnerability [42,43,44,45].

Single-nucleus RNA sequencing (snRNA-seq) has revolutionized the resolution of the cellular landscape in the AD brain [46,47,48,49,50], having discovered the disease-associated microglia (DAM) [51,52,53], an activated state localized to Aβ plaques and implicated in clearance, and the disease-associated astrocyte (DAA), a reactive astrocytic population involved in neuroinflammatory responses and synaptic dysfunction [54,55,56]. These discoveries underscore that snRNA-seq is a powerful tool not only for mapping cell states but also for investigating the cellular coordination governing AD pathology through integration of data across brain regions and capture of coordinated glial–neuronal states.

Microglia exhibit remarkable heterogeneity across different brain regions. Research indicates that there are significant differences in the highly expressed genes of microglia in the prefrontal cortex (PFC), cerebellum, and hippocampus (HPC) of AD patients, and the microglia in AD patients comprise multiple functionally distinct subtypes [53,57,58,59,60]. The different distribution of microglial subtypes across brain regions may help explain the selective vulnerability of AD pathology. These findings suggest the value of integrating snRNA-seq data to elucidate cell-type-specific responses and intercellular communication networks in AD.

This study aims to investigate microglia–neuron crosstalk in the human AD brain, with a particular focus on region-specific alterations in microglia and their interactions with neurons. By employing pseudotime trajectory inference and cell–cell communication analysis, we seek to characterize the transcriptional variations within microglia and their dynamic interplay with neurons. We anticipate that this approach will reveal patterns of dysregulated cellular communication across different AD brain regions, thereby providing new insights for the discovery of early diagnostic biomarkers and the development of targeted therapeutic strategies.

## 2. Results

### 2.1. snRNA-Seq Analysis of Occipital Lobe, Prefrontal Cortex, and Hippocampus upon AD

**Figure 1:**
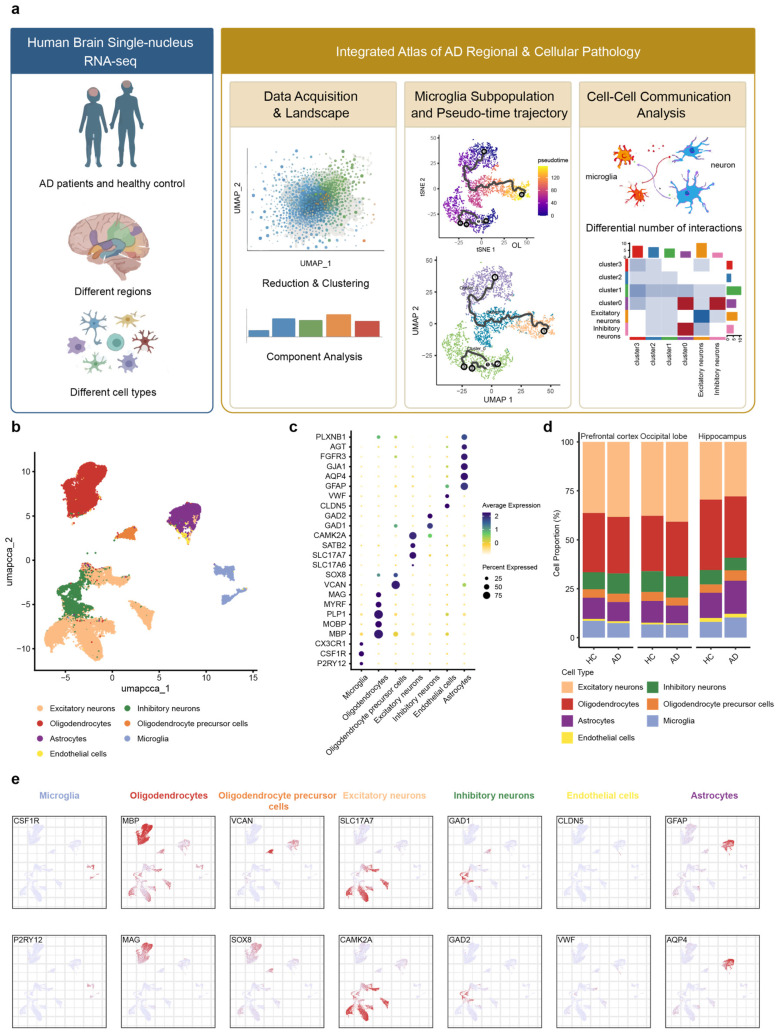
snRNA-Seq analysis of three brain regions in AD patients and healthy Control. (**a**) Based on the predefined inclusion criteria, snRNA-seq datasets of brain tissues from Alzheimer’s disease patients and HC were screened and obtained from the GEO database for this study. (**b**) UMAP of snRNA-Seq data showing seven cell clusters from 14 patients’ brain tissue. (**c**) Dot plot of the marker gene of each cell type. (**d**) Proportion changes of seven cell types across brain regions in AD patient and HC. (**e**) Feature plot of the marker gene of each cell type. Each column in the feature plot represents a cell type, with two representative genes selected for each cell type.

We downloaded three datasets from publicly accessible repositories. These included snRNA-seq data derived from the occipital lobe, prefrontal cortex, and hippocampus of post-mortem human brains, which were obtained from the ssREAD database (https://bmblx.bmi.osumc.edu/ssread/, accessed on 20 October 2025), under accession numbers AD013, AD024, and AD046 (details see in method and Table 1). A stringent quality control was applied, retaining only high-quality cells that expressed between 200 and 5000 genes and exhibited a mitochondrial gene content below 10%. Through this pipeline, we obtained an integrated dataset comprising 14 individuals and a total of 78,845 high-quality cells, which was subsequently used for downstream clustering and in-depth bioinformatic analysis (Figure 1a).

Based on the expression of marker genes derived from the ssREAD database [61], the remaining cells were annotated into seven main cell types (Figure 1b): microglia (P2RY12, CSF1R), astrocytes (GFAP, AQP4), oligodendrocytes (MBP, MOBP), oligodendrocyte precursor cells (VCAN, SOX8), excitatory neuron (SLC17A6, SLC17A7), inhibitory neurons (GAD1, GAD2), and endothelial cells (CLDN5, VWF). The expression of representative cell markers was shown by dot plot (Figure 1c,e), confirming our accuracy of the cluster identity.

Further analysis of cell type proportions across the occipital lobe, prefrontal cortex, and hippocampus revealed the alterations in microglial and astrocytic populations in AD patients compared to HC (healthy control) (Figure 1d). In the prefrontal cortex, both microglia and astrocyte proportions were decreased. Conversely, the hippocampus exhibited an increase in both cell types. Within the occipital lobe, microglial proportions remained unchanged, while astrocytes decreased. These findings revealed that microglia and astrocytes exhibit synchronous or differential patterns of change across distinct brain regions in AD, collectively highlighting the regional specificity of disease progression.

### 2.2. Microglia Display Predominant Transcriptomic Perturbations and Functional Divergence in AD

**Figure 2:**
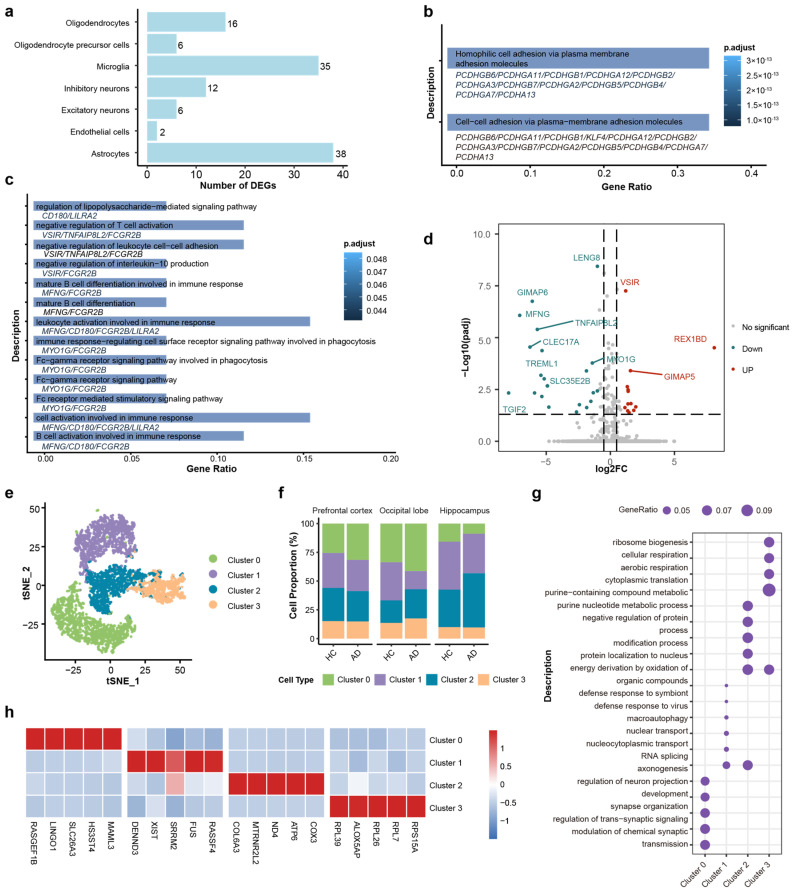
DEGs and functional enrichment analysis of microglia cells and subtypes. (**a**) Bar plot quantifying the number of differentially expressed genes (DEGs) across major cell types. Microglia and astrocytes show the highest burden of transcriptomic changes. (**b**) GO enrichment analysis of astrocytic DEGs. (**c**) GO enrichment analysis of microglial DEGs. (**d**) Volcano plot of DEGs in microglia (AD vs. HC). Red and blue dots indicate significantly upregulated and downregulated genes, respectively (FDR < 0.05). (**e**) t-SNE visualization of four distinct microglial sub-clusters (Clusters 0–3). (**f**) Stacked bar charts showing the relative proportions of microglial sub-clusters across the different brain regions in HC and AD groups. (**g**) Dot plot of the top enriched biological processes for each sub-cluster. (**h**) Heatmap of the top 5 marker genes for each sub-cluster. Color scale indicates relative expression.

To understand the functional alterations in the brains of AD patients, differentially expressed genes (DEGs) of each cell type were calculated by using thresholds of |log2(Fold Change)| > 1 and false discovery rate (FDR) < 0.05. Microglia and astrocytes exhibited the most profound transcriptomic alterations, as evidenced by their significantly higher counts of DEGs compared to others (Figure 2a). Collectively, these findings indicate that glial cells, particularly microglia and astrocytes, play an important role in the transcriptional dysregulation observed in Alzheimer’s disease.

GO analysis shows that the primary functional alterations in astrocytes were relatively homogeneous and predominantly enriched in cell–cell adhesion via plasma–membrane adhesion molecules (Figure 2b). However, the DEGs of microglia were significantly enriched in several key pathways related to immune response, including lipopolysaccharide-mediated signaling (which activates antigen-presenting cells and bridges innate and adaptive immunity [62,63]), Fc-gamma receptor pathways (associated with pathogen phagocytosis and clearance [64]), and regulation of leukocyte cell–cell adhesion (involved in immune cell migration and interaction with antigen-presenting cells [65,66]) (Figure 2c). The volcano plot showed the DEGs in microglia between the AD group and HC group, including 13 upregulated genes and 22 downregulated genes (Figure 2d). The upregulated genes, such as VSIR (V-set immunoregulatory receptor) and GIMAP5 (GTPase of immunity associated protein 5), and the downregulated genes, such as GIMAP6 (GTPase of immunity associated protein 6), MFNG (MFNG O-fucosylpeptide 3-beta-N-acetylglucosaminyltransferase), and TGIF2 (TGFB induced factor homeobox 2), are functionally associated with immune regulation. VSIR has been established as a negative immune checkpoint molecule [67,68]. The GIMAP family genes were significantly associated with immune cell infiltration and immune checkpoint molecules [69]. MENG regulated T cell development by enhancing the interactions of Notch signaling [70], and TGIF2 expression may modulated the activation status of microglia [71]. These studies suggest that the immune function of microglia may have been significantly altered in the AD process.

AD is characterized by significant pathological transformations in microglia, which collectively drive neurodegenerative progression through intricate intercellular interactions [72,73,74,75]. Given the critical function in AD and the diversity of microglial GO functional changes, we selected microglia as the primary focus for further study, even though astrocytes exhibited a greater number of differentially expressed genes.

Next, we employed further classification for microglia cells. Based on single-cell transcriptomic profiles, we identified four distinct microglial subtypes (clusters 0–3) through unsupervised clustering. Four subtypes were identified (Figure 2e), and the cell proportions across three brain regions are shown in Figure 2f. The distinct functional identities of the microglial subtypes, as defined by their unique marker genes (Figure 2g), were further highlighted by GO pathway analysis (Figure 2h): Cluster 0 was characterized by high expression of LINGO1, retained a homeostatic signature enriched in synapse organization, while Cluster 1 was associated with RNA splicing and nuclear transport (e.g., XIST, SRRM2). Cluster 2 was defined by high expression of metabolism associated genes (e.g., COX), suggestive of heightened metabolic activity. Cluster 3 was characterized by ribosomal protein expression (e.g., RPL39) and ribosome biogenesis, indicating high translational activity.

### 2.3. Pseudotime Trajectory Reveals the Dynamic Transition and Functional Dysregulation of Microglial States in AD

**Figure 3:**
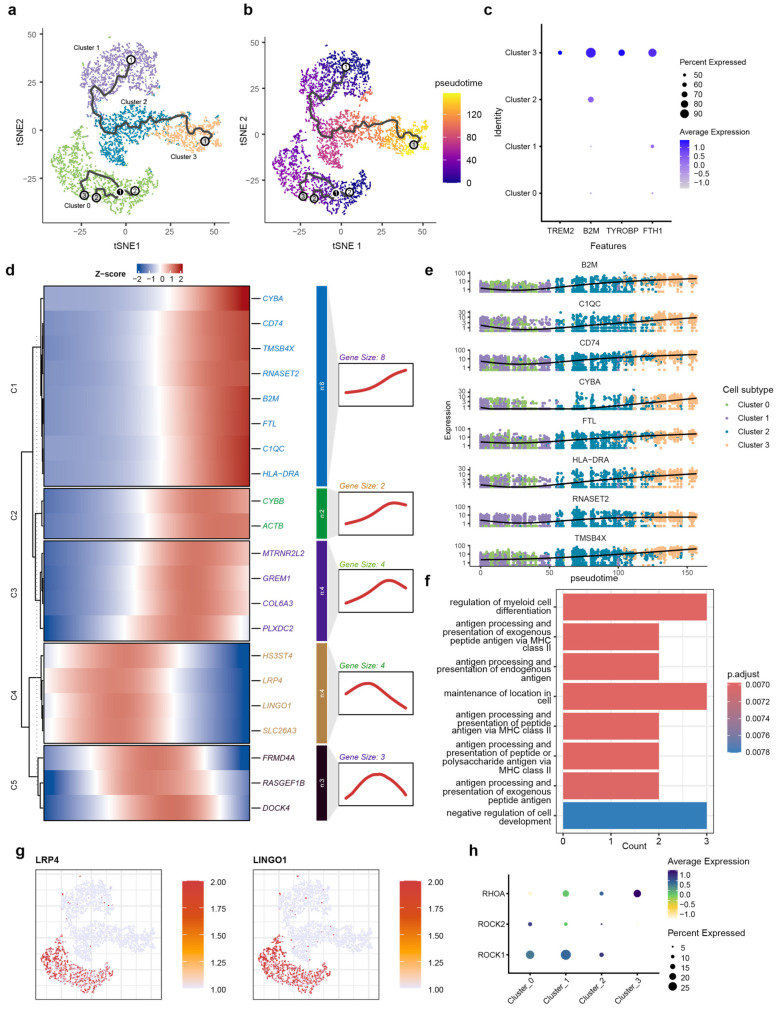
Pseudotime trajectory and kinetic transcriptional profiling of microglial transition. (**a**) Schematic diagram of the pseudotime analysis workflow; (**b**) t-SNE visualization of microglial subtypes (0–3) superimposed with the inferred trajectory path, demonstrating the transition from Cluster 0 toward terminal branches. Root (white), Branch (black) and Leaf nodes (gray) indicate starting points, points where the trajectory splits, and the endpoints of the trajectory, respectively. The labels 1, 2, and 3 are used to distinguish between the different cellular states shown. (**c**) t-SNE projection of microglia colored by pseudotime, showing a progressive transition from the root (dark purple) to terminal states (yellow). (**d**) Heatmap showing the pseudo-temporal expression dynamics of significant DEGs, categorized into five kinetic modules (C1–C5) based on their expression patterns along the trajectory. (**e**) Trajectory plots of representative genes from module C1, showing a decrease in expression as pseudotime increases. (**f**) GO enrichment analysis of C1 module genes, highlighting biological processes such as antigen processing and presentation and myeloid cell differentiation. (**g**) Feature plots showing the cluster-specific expression of LRP4 and LINGO1, predominantly localized to homeostatic Cluster 0. (**h**) Bubble plot visualizing the expression of LINGO1-RhoA-ROCK pathway components across microglial clusters. The dot size represents the percentage of cells expressing the gene, and the color intensity reflects the average expression level, indicating specialized signaling activity in Cluster 0.

To reconstruct the dynamic, continuous processes of cellular state transitions for microglia subtypes, we employed pseudotime analysis, which infers a continuous trajectory from single-cell transcriptomes, thereby revealing the potential sequence of transcriptional changes microglia undergo during AD pathogenesis. Pseudotime analysis was performed using Monocle3 (v1.3.4) on microglia subsets isolated from integrated snRNA-seq data. The trajectory originated from Cluster 1, transited through an intermediate state in Cluster 2, and terminated in Cluster 3 (Figure 3a,b). Cluster 0 was mapped to an isolated position. Notably, based on its high expression of relevant marker genes, Cluster 3 was identified as DAM cells. The marker gene for Cluster 3 is the DAM marker obtained from published studies (Figure 3c). Next, we identified genes that were dynamically regulated throughout the differentiation process. The heatmap displays dynamic gene modules along the pseudotime trajectory, classified into five distinct modules (C1–C5) based on their expression profiles (Figure 3d).

Genes in module C1 are highly expressed toward the pseudotime terminus. This module is highly enriched in core AD-associated markers, including TREM2, TYROBP, APOE, and HLA-DRA, and the synchronized upregulation of CYBA, C1QC, and B2M suggest that at the trajectory’s end, microglia have completely transitioned into the DAM state (Figure 3e). GO analysis showed that genes in module C1 were enriched for MHC class II-dependent antigen processing and presentation pathways, also suggesting the functional transition to immune-activated state (Figure 3f).

Pseudotime trajectory also revealed another category of gene (C2 and C3) exhibiting a pattern of initial increase followed by a decrease along the pseudotime terminus. Genes associated with immune activation (CYBB) and cytoskeletal reorganization (ACTB) were enriched in module C2, and genes related to endogenous neuroprotection (MTRNR2L2), pro-inflammation differentiation of microglia (GREM1) extracellular matrix remodeling (COL6A3), and vascular response (PLXDC2) were enriched in module C3. The expression of gene modules C2 and C3 suggest that under the pathological background of AD, microglia undergo a sustained activation process, involving the coordinated activation of various functions, including endogenous neuroprotection, pro-inflammatory signaling, extracellular matrix remodeling, and vascular responses. However, this activated state does not persist indefinitely.

The expression patterns of C4 and C5 indicate that the function of these two modules are not representative of a terminal state of microglia but rather a transitional phase. The C4 module primarily encompasses genes functionally associated with the extracellular matrix (HS3ST4), neuronal activity and intracellular signal transduction (LRP4, LINGO1), and inflammation (SLC26A3). The C5 module consists mainly of genes related to the cytoskeleton (FRMD4A, RASGEF1B, and DOCK4). These biological processes are likely to influence the activation and migration of microglia.

We also observed that genes within modules C4 and C5 were progressively downregulated along the pseudotime trajectory. LINGO1, a key marker of C4, has been implicated as a brake on microglial activation [76,77]. This finding suggests that the downregulation of LINGO1 may facilitate microglial activation during the differentiation process, potentially contributing to neuroinflammatory responses.

LINGO1 and LRP4 were also identified as key markers specifically enriched in the Cluster 0 population (Figure 3g). The high expression of LINGO1 and its associated signaling components—including RHOA, ROCK1, and ROCK2—suggests a specific functional constraint within this subtype [78,79,80,81] (Figure 3h). This transcriptional profile indicates the existence of a distinct microglial subpopulation with elevated LINGO1 expression. LINGO 1 can interact with the Nogo-signaling system [75], which further activates RhoA and ROCK1/ROCK2 to restrict neurons and is involved in neuroinflammation through the regulation of microglial inflammatory activation [82,83]. Research indicates that inhibiting LINGO-1 with specific antibodies increases microglial numbers in APP/PS1 mice [84]. Therefore, the high expression of LINGO-1 in Cluster 0 microglia likely characterize an inhibitory state that restrains their phenotypic transition from a homeostatic to a disease-associated state.

### 2.4. Disrupted Microglia–Neuron Crosstalk Across AD Brain Regions

**Figure 4:**
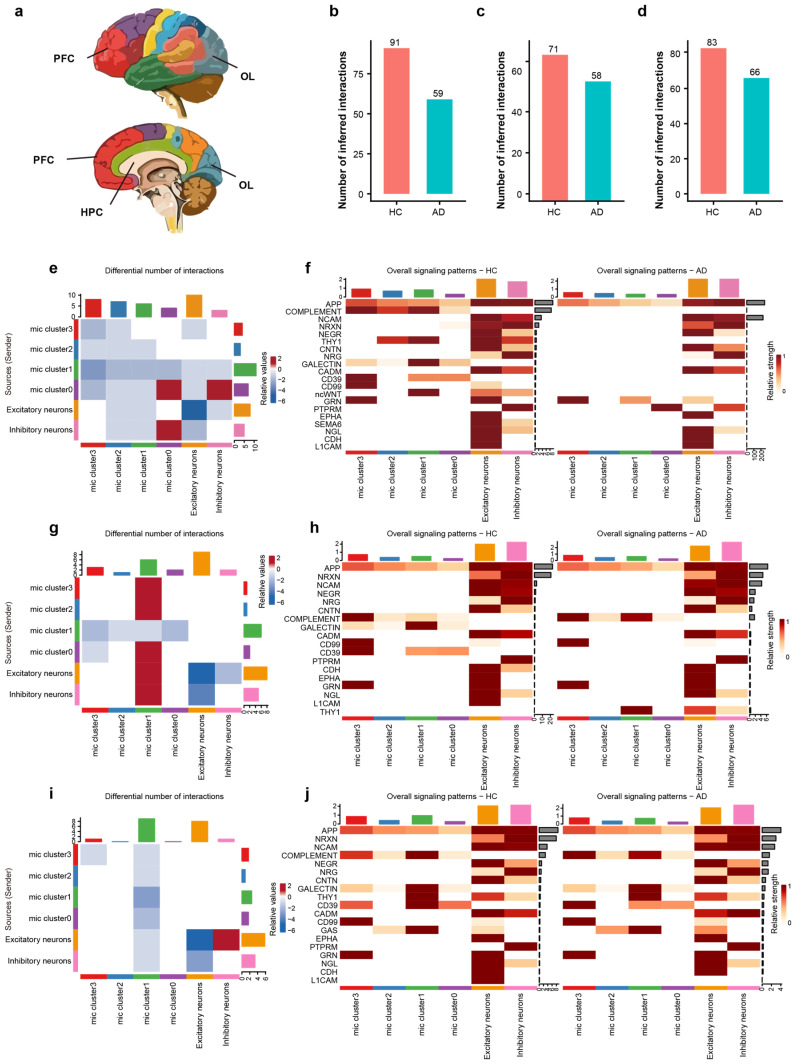
Regional and sub-cluster specific disruption of microglia–neuron crosstalk in AD. (**a**) Schematic diagram illustrating the anatomical locations of the analyzed brain regions: hippocampus, prefrontal cortex, and occipital lobe. Colors correspond to different brain regions. (**b**–**d**) Bar plots showing the total number of inferred interactions in HC vs. AD for the HPC (**b**), PFC (**c**), and OL (**d**), highlighting a global decrease in communication in AD. (**e**) Differential interaction heatmap in the HPC, visualizing the change in interaction strength between microglial sub-clusters and neurons (Red: increased in AD; Blue: decreased in AD). (**f**) Heatmap showing the overall signaling flow of the top 20 signaling pathways in the hippocampus across microglial clusters and neurons between AD and HC. (**g**) Differential interaction heatmap in the prefrontal cortex. (**h**) Heatmap showing the relative incoming and outgoing signaling flow of the top 20 signaling pathways in the PFC between AD and HC. (**i**) Differential interaction heatmap in the OL, revealing region-specific communication perturbations. (**j**) Heatmap showing the relative incoming and outgoing signaling flow of the top 20 signaling pathways in the occipital lobe between AD and HC.

To examine region-specific intercellular communication, we used CellChat to compare dysregulated communication patterns of microglia in the prefrontal cortex (PFC), hippocampus (HPC), and occipital lobe (OL) between HC and AD brains (Figure 4a). The analysis was performed using microglia and neuron subsets isolated from integrated snRNA-seq data. Analysis revealed a consistent and significant reduction in the total number of inferred intercellular interactions in AD brains relative to HC across all examined regions (Figure 4b–d), suggesting that the microglia–neuron crosstalk is significantly compromised in the AD brain, indicating an important role in AD progression.

We characterized the functional alterations in cellular signaling networks in AD, delineating signal sources/senders and targets/receivers in the HPC (Figure 4e). Among all cell populations, Cluster 0 exhibited the most pronounced differential interaction counts than others. In detail, Cluster 0 showed strong bidirectional interactions with inhibitory neurons, both as a sender and a receiver, indicating that the inhibitory neurons and Cluster 0 are critical for hippocampal microglia–neuron crosstalk in AD.

Beyond quantifying the overall communication number, our analysis delineated 20 signaling pathways to decipher critical intercellular signals among diverse cell populations, including APP, complement, NCAM, NRXN, NEGR, THY1, CD39, CD99, and L1CAM etc. We specifically identified individual signaling pathways operating between microglia and neurons (Figure 4f). Notably, compared to the HC group, Cluster 0 exhibited a significant increase in the relative signaling flow of PTPRM alongside a decrease in CD39 signaling. Consequently, we infer that PTPRM signaling plays a pivotal role within Cluster 0 during the AD process.

We characterized the functional alterations in cellular signaling networks within the PFC of AD, detailing signal senders and receivers. As a receiver, Cluster 1 demonstrated robust interactions with Cluster 0, Cluster 2, Cluster 3, as well as with both excitatory and inhibitory neurons (Figure 4g). Analysis of signaling pathways revealed that the complement and THY1 pathways were significantly enriched in Cluster 1 of the AD group (Figure 4h), suggesting that THY1 and the complement pathway are key molecules underlying the altered communication in PFC.

The OL exhibited changes distinct from those in the PFC and HPC, characterized by a reduction in communication strength within Cluster 1 (Figure 4i). Specifically, our signaling pathway analysis in the OL did not detect any pathways that were significantly enriched in AD group’s Cluster 1 (Figure 4j). These findings underscore the heterogeneity of microglia–neuron crosstalk in AD, with the HPC and PFC showing distinct signaling pathway activations associated with specific microglial subpopulations, while the OL follows a different adaptive or degenerative trajectory.

### 2.5. Aberrant Microglia–Neuron Crosstalk Is Mediated by Region-Specific Ligand–Receptor Pairs in the Prefrontal Cortex, Hippocampus, and Occipital Lobe of AD

**Figure 5:**
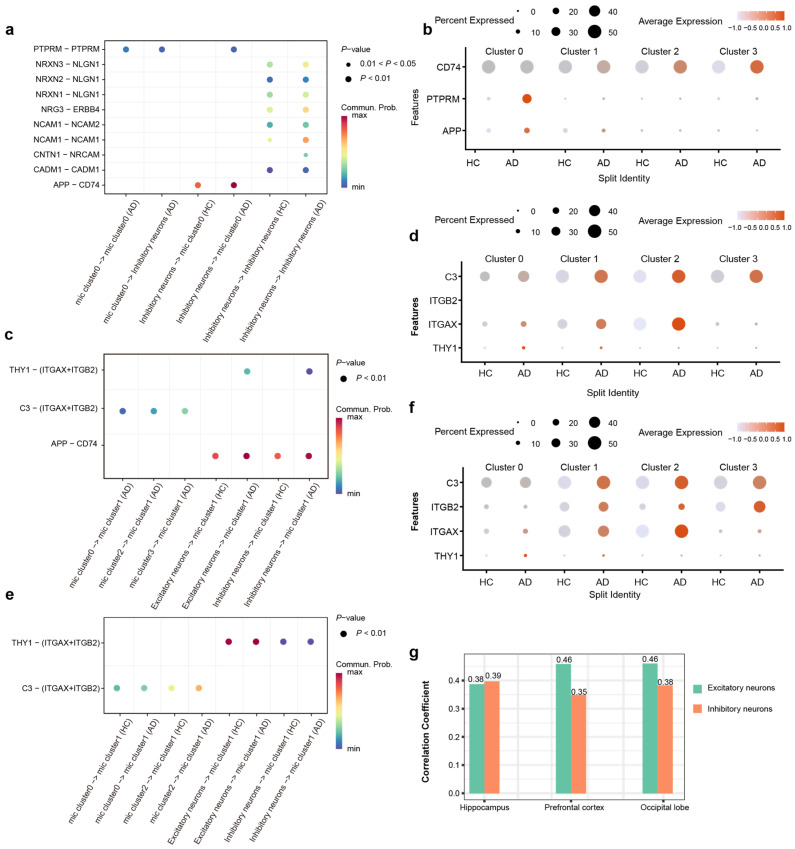
Region-resolved analysis reveals distinct microglia–neuron crosstalk landscapes in the AD brain. (**a**) Bubble plot shows the significantly differential ligand–receptor interactions involving Cluster 0 microglia in the HPC, highlighting the amplification of PTPRM and APP-CD74 signaling axes. (**b**) The ligand–receptor pairs involved in the PTPRM and APP-CD74 signaling axes were differentially expressed in HC and AD within the HPC. (**c**) Bubble plot shows the significantly differential ligand–receptor interactions involving Cluster 1 microglia in the PFC, highlighting the amplification of C3 and THY1 signaling axes. (**d**) The ligand–receptor pairs involved in the C3-Integrin and THY1-Integrin signaling axes were differentially expressed in HC and AD within the PFC. (**e**) Bubble plot shows the significantly differential ligand–receptor interactions involving Cluster 1 microglia in the OL, highlighting the amplification of C3 and THY1 signaling axes. (**f**) The ligand–receptor pairs involved in the C3-Integrin and THY1-Integrin signaling axes were differentially expressed in HC and AD within the OL. (**g**) Correlation analysis of THY1 with AD risk in HPC PFC and OL.

Our analysis of cell–cell communication across PFC, HPC, and OL reveals that the observed alterations in communication strength between specific microglial subtypes and neurons are driven by distinct molecular signaling axes. In the HPC, we observed a significant upregulation of the APP-CD74 interaction specifically between inhibitory neurons and Cluster 0 microglia (Figure 5a). Corroborating this aberrant interaction, the expression levels of APP, PTPRM, and CD74 were significantly increased in AD patients compared to healthy controls (Figure 5b). These findings suggest that PTPRM-mediated adhesion and APP-CD74 signaling constitute hippocampus-specific modalities of pathological crosstalk between microglia and inhibitory neurons, likely emerging in response to AD pathology.

In PFC, we observed that the enhancement of microglia–neuron crosstalk was predominantly driven by Cluster 1 microglia. Further interrogation of ligand–receptor interaction strengths revealed a marked amplification of the C3-(ITGAX + ITGB2) and THY1-(ITGAX + ITGB2) signaling axes in AD patients. Then, we detected a significant upregulation of C3, ITGAX, and THY1 expression in the AD patients compared to HC (Figure 5c). These findings suggest that the region-specific remodeling of Cluster 1 microglial communication patterns in the PFC of AD may be fundamentally mediated by the dysregulation of complement-integrin and THY1-integrin signaling pathways (Figure 5d).

Cluster 1 microglia in the OL region exhibited a decline in their interaction network (Figure 4i). Paradoxically, this reduction in intercellular communication was accompanied by a significant transcriptional upregulation of genes encoding critical interaction pairs, such as C3, ITGAX, ITGB2, and THY1, in AD patients (Figure 5e,f). The increased expression of these molecules did not translate into a higher number of detectable interactions, pointing to potential post-transcriptional or functional impairments in the AD context. The divergent intensity of microglia–neuron communication likely mirrors the region-specific heterogeneity of AD pathology. This finding underscores that such interactions are intricately linked to the unique pathological microenvironment and functional specialization of distinct brain regions.

Intriguingly, our analysis revealed a specific association between THY1 and AD. Specifically, THY1 expression in excitatory and inhibitory neurons across the HPC, PFC, and OL was significantly correlated with AD pathology, a pattern not observed for other genes (Figure 5g). Notably, this correlation was most pronounced in the excitatory neurons of the PFC and OL. Collectively, our findings imply that regional variations in the abundance of neuronal surface ligands regulate local microglia–neuron crosstalk, thereby contributing to the differential regional susceptibility to AD pathogenesis.

## 3. Discussion

By integrating pseudotime trajectory inference with intercellular communication analysis, this study delineated heterogeneity across glial subtypes and brain regions, uncovering the dynamic evolution of microglia in AD and identifying potential key mediators of microglia–neuron crosstalk.

We first defined four clusters based on distinct functional enrichment profiles, suggesting the complex landscape of microglial heterogeneity. Cluster 0 exhibited high expression of LINGO1, a gene linked to multiple neurodegenerative diseases including AD [84]. LINGO1 interacts with downstream effectors such as RhoA, WNK, and PI3K to regulate neuronal survival and axon regeneration. Antagonizing LINGO1 reduces microglial activation and improves cognitive impairment in APP/PS1 mice [85]. Cluster 1 and Cluster 2 appear as intermediated states, showing upregulation of stress-response markers and COX subunits. These clusters appear to serve as a transitional phase before microglia commit to a fully reactive phenotype and also adapt to sustain inflammatory in AD environment. We also found that TYROBP was a highly expressed gene in Cluster 3 (Supplementary Table S1), which has been promoted as a marker of microglial transition from a homeostatic to a disease-associated state [86]. As a recognized marker gene for DAM [51,52], previous studies have shown that microglia with high TYROBP expression are involved in phagocytosing Aβ plaques [87]. Therefore, we speculate that Cluster 3 likely represents morphologically activated DAM that aggregate around Aβ plaques in the AD brain. This transformation indicates that microglia transition from a homeostatic state to a more active inflammatory state in response to AD pathology. While microglia disrupt this immune balance, it further exacerbates pathological damage in AD.

Pseudotime analysis delineates a clear evolutionary continuum for microglia, originating from homeostatic Cluster 1 and advancing toward specialized reactive states in Clusters 2 and 3. Dynamics of significant DEGs were categorized into five kinetic modules (C1–C5), and we observed that module C1 and modules C4/C5 showed opposing yet interconnected expression changes over pseudotime. They form a regulatory network that drives microglia toward a disease-associated state. Along this progression, LINGO1 was significantly downregulated, while genes such as HLA, CYBA, and CD74 were progressively upregulated. Upregulation of module C1 indicates the acquisition of core DAM features and MHC class II antigen presentation pathways, reflecting a shift toward an immune-activated end state. In contrast, downregulation of modules C4 and C5, especially the lower expression of the key inhibitor LINGO1, likely removes an intrinsic brake on microglial activation. These modules reveal an internal molecular switch, where microglia may transition into a pro-inflammatory, neurotoxic DAM phenotype by simultaneously downregulating homeostatic modules and upregulating immune effector modules.

We also observed regional heterogeneity in microglia–neuron crosstalk across the HPC, PFC, and OL. Microglial spatial heterogeneity remains a crucial yet not fully answered question in the context of potential cell-directed therapies for AD [88]. Two principal factors may explain this regional variation. One is the diversity in microglial activation and gene expression among different brain regions, which cause different responses to injury, disease or inflammatory challenge and also vary according to neuroaxis location [89]. The other relates to the selective susceptibility of certain brain regions in AD, wherein localized pathological changes may, in turn, drive spatially heterogeneous microglia–neuron interactions [90,91]. This may explain why we infer that PTPRM signaling plays a pivotal role especially in HPC, and the complement and THY1 pathways were significantly enriched in PFC.

Despite regional differences in microglial activation, the PFC and OL shared a common molecular mechanism in microglia–neuron crosstalk: a THY1-dependent response pattern. THY1, a kind of immunoglobulin protein which is involved in cell adhesion and cell communication in numerous cell types, but particularly in cells of the immune and nervous systems [92,93], mediates intercellular interactions by engaging the microglial receptors ITGB2 and ITGAX, two microglial receptors belonging to the integrin family. The integrin family are involved in microglial phagocytosis in the central nervous system and contribute to the progression of pathology and cognitive decline in AD [94]. Our data showed elevated expression of THY1, ITGAX, and ITGB2 in the PFC and OL, suggesting that heightened regional inflammation coincides with altered microglia–neuron communication via the THY1-ITGAX/ITGB2 axis.

Conversely, the most prominent signaling change in the HPC occurred in PTPRM, a tyrosine phosphatase involved in diverse cellular processes including growth, differentiation, and mitotic regulation. Conversely, the most prominent signaling change in the HPC occurred in PTPRM, a tyrosine phosphatase involved in diverse cellular processes including growth, differentiation, and mitotic regulation. Protein tyrosine phosphatases (PTPs) are important regulators of neuronal signal transduction. A growing number of PTPs have been implicated in AD, where they contribute to intracellular accumulation of Aβ and tau and are involved in regulating synaptic plasticity and stress responses [95]. Together, the region-specific ligand–receptor alterations reflect distinct molecular mechanisms that likely contribute to spatially selective vulnerabilities in AD pathogenesis.

Notably, although THY1 signaling strength did not increase significantly in the HPC, THY1 expression still correlated strongly with AD pathology across the HPC, PFC, and OL. This pattern suggests that HPC may being uniquely vulnerable in early AD and may operate within a narrower homeostatic range. Even a subtle dysregulation of the THY1 pathway, below the threshold of a significant change, could be sufficient to disrupt its finely tuned microglia–neuron crosstalk, thereby yielding the strongest correlation with disease progression in this region.

Above all, this study delineates a dynamic landscape of microglial involvement in AD. Our analysis showed that microglia undergo a coordinated transform into a disease-associated state, governed by inhibitory modules (e.g., LINGO1) while upregulating inflammatory and antigen-presenting pathways. This transition manifests with regional heterogeneity, as the PFC and OL engage a THY1-dependent communication axis, whereas the HPC relies on PTPRM signaling and exhibits unique vulnerability to subtle THY1 dysregulation. Our findings propose that AD progression is fueled not by a uniform glial response but by regional heterogeneity in microglia–neuron crosstalk, offering a refined framework for understanding spatial selectivity in pathology and for developing targeted therapeutic strategies.

## 4. Materials and Methods

### 4.1. Data Sources and Workflow

snRNA-seq data from human post-mortem brain tissues were obtained from the ssREAD database (https://bmblx.bmi.osumc.edu/ssread/, accessed on 20 October 2025), a comprehensive resource integrating transcriptomic data for AD research. The dataset utilized in this study comprised 14 human-specific samples derived from multiple brain regions, including the hippocampus, entorhinal cortex, and prefrontal cortex, with detailed annotations for disease status (AD vs. HC). Raw gene expression matrices were subjected to rigorous quality control using Seurat (v5.4.0) to remove low-quality cells, retaining those with detected gene counts between 200 and 5000 per cell and mitochondrial gene content below 10%. Expression data were normalized, and technical batch effects were corrected using Canonical Correlation Analysis (CCA) integration to ensure robust cross-sample comparability. The FindVariableFeatures function in Seurat was employed to identify the top 2000 highly variable genes. The expression levels of these genes were scaled prior to dimensionality reduction. Principal component analysis was performed, and the top 30 principal components were used for downstream clustering and cell population identification. A total of seven distinct cell clusters were identified using a graph-based clustering algorithm with a resolution parameter of 1.0.

Cell type identities were annotated based on the expression of canonical lineage markers identified via differential expression analysis (Wilcoxon rank-sum test; *p* < 0.05). Major cell lineages (e.g., excitatory neurons, inhibitory neurons, microglia, oligodendrocytes, astrocytes, oligodendrocyte precursor cells, endothelial cells) were defined using established markers in Figure 1e. To further dissect microglial heterogeneity, the microglia cluster was subset and subjected to a second round of high-resolution clustering. This iterative approach allowed for the identification of fine-grained microglial subpopulations (Clusters 0–3), which were subsequently characterized by distinct functional gene signatures and gene ontology (GO) pathway enrichment.

### 4.2. Identification of the Differentially Expressed Genes (DEGs) and GO Enrichment Analysis

A function of Seurat called FindMarker was used to identify the DEGs of each cell type between the healthy control and AD group according to the following threshold criteria: (1) average log2(fold change) ≥ 1, (2) *p* < 0.05. We used a volcano plot to visualize the DEGs. After DEGs filtering, GO enrichment analysis with the org.Mm.eg.db database and enrichGO function by the ClusterProfiler software (v 4.6.2) was utilized to reveal the main functional alteration of the cell type. The Benjamini–Hochberg (BH) method was used for the multiple test adjustments. Further, ggplot2 (v 3.4.2) was used for visualization.

### 4.3. Pseudotime Analysis

Pseudotime analysis was performed using Monocle3 (v1.0.1) to reconstruct continuous trajectories of cellular state transitions from snRNA data. Initially, the expression matrix and cell metadata were extracted from a quality-controlled and annotated Seurat object and converted into a Monocle3-compatible cell_data_set object. Dimensionality reduction was then carried out using the reduce_dimensionfunction to project the data into a TSNE space. The developmental trajectory was constructed using the learn_graph function, which models the progression path based on cellular similarities within the reduced-dimensional space. Pseudotime values were computed with the order_cells function, designating an early cell population as the trajectory root based on biological prior knowledge, thereby assigning each cell a relative temporal position along the inferred path. To validate trajectory reliability, the correlation between pseudotime and the expression of key genes was further analyzed.

### 4.4. Cell–Cell Communication Analysis

Cellular communication networks were inferred using the CellChat package (v1.6.1), which leverages ligand–receptor interaction databases to quantify intercellular signaling probabilities. The analysis focused on interactions involving excitatory neurons, inhibitory neurons, and microglial subtypes, with signaling pathways deemed significant if their interaction strength exceeded random permutations (*p* < 0.05). We quantified the communication probability for each ligand–receptor pair using the law of mass action, identifying significant interactions via a permutation test. To identify AD-associated signaling perturbations, we performed differential communication analysis, isolating ligand–receptor axes, such as THY1-Integrin and PTPRM, that exhibited significantly altered information flow in the AD condition compared to HC.

### 4.5. Correlation Analysis with AD Pathology

Associations between molecular signatures and AD pathology were assessed using Pearson correlation analysis. Genes exhibiting robust monotonic relationships with disease severity were prioritized based on statistical significance (*p* < 0.05) and a correlation coefficient threshold of |ρ| > 0.3.

## Figures and Tables

**Table 1:** Demographic information and sample identifiers of snRNA-seq cohorts.

Data ID	Species	Condition	Region	Public ID
AD02405	Human	AD	Occipital lobe	GSE148822
AD02406	Human	AD	Occipital lobe	GSE148822
AD01303	Human	AD	Prefrontal cortex	GSE157827
AD01304	Human	AD	Prefrontal cortex	GSE157827
AD04602	Human	AD	Hippocampus	GSE199243
AD04604	Human	AD	Hippocampus	GSE199243
AD02401	Human	HC	Occipital lobe	GSE148822
AD02402	Human	HC	Occipital lobe	GSE148822
AD02403	Human	HC	Occipital lobe	GSE148822
AD02404	Human	HC	Occipital lobe	GSE148822
AD01301	Human	HC	Prefrontal cortex	GSE157827
AD01302	Human	HC	Prefrontal cortex	GSE157827
AD04601	Human	HC	Hippocampus	GSE199243
AD04603	Human	HC	Hippocampus	GSE199243

The cohort consisted of Alzheimer’s disease (AD) patients and healthy control (HC) donors. The sample identifiers provided allow for the precise retrieval of sequencing data from public repositories.

## Data Availability

The original data presented in the study are openly available in the ssREAD dataset (https://bmblx.bmi.osumc.edu/ssread/, accessed on 20 October 2025). The accession numbers are AD013, AD024, and AD046.

## Supplementary Materials

The following supporting information can be downloaded at: https://www.mdpi.com/article/10.3390/ijms27031492/s1.

## Abbreviations

The following abbreviations are used in this manuscript:

AD	Alzheimer’s disease
PFC	Prefrontal cortex
HPC	Hippocampus
OL	Occipital lobe
DAM	Disease-associated microglia
DAA	Disease-associated astrocytes
SnRNA-seq	Single-nucleus RNA sequencing
HC	Healthy control
DEGs	Differentially expressed genes
FC	Fold Change
FDR	False discovery rate
GO	Gene ontology
VSIR	V-Set Immunoregulatory Receptor
GIMAP5	GTPase IMAP Family Member 5
GIMAP6	GTPase IMAP Family Member 6
MFNG	O-Fucosylpeptide 3-Beta-N-Acetylglucosaminyltransferase
TGIF2	Transcription Growth Factor-Beta-Induced Factor 2
LINGO1	Leucine rich repeat and Ig domain containing 1
Nogo	Neurite outgrowth
ITGB2	Integrin Subunit Beta 2
ITGAX	Integrin Subunit Alpha X
Thy1	Thy-1 Cell Surface Antigen
PTPs	Protein tyrosine phosphatases
PTPRM	Protein Tyrosine Phosphatase Receptor Type M
